# The 2021 update of the EPA’s adverse outcome pathway database

**DOI:** 10.1038/s41597-021-00962-3

**Published:** 2021-07-12

**Authors:** Holly M. Mortensen, Jonathan Senn, Trevor Levey, Phillip Langley, Antony J. Williams

**Affiliations:** 1 Center for Public Health and Environmental Assessment, Office of Research and Development, U.S. Environmental Protection Agency, 109 T.W. Alexander Dr., Research Triangle Park, NC 27709 USA; 2 Oak Ridge Associated Universities, Research Triangle Park, NC 27709 USA; 3 Present Address: SAS Institute 100 SAS Campus Dr, Cary, NC 27513 USA; 4 Present Address: International Farming Corporation, LLC, 1318 Dale St, Raleigh, NC 27605 USA; 5 Center for Computational Toxicology and Exposure, Office of Research and Development, U.S. Environmental Protection Agency, 109 T.W. Alexander Dr., Research Triangle Park, NC 27709 USA

**Keywords:** Data integration, Genetic databases, Protein databases

## Abstract

The EPA developed the Adverse Outcome Pathway Database (AOP-DB) to better characterize adverse outcomes of toxicological interest that are relevant to human health and the environment. Here we present the most recent version of the EPA Adverse Outcome Pathway Database (AOP-DB), version 2. AOP-DB v.2 introduces several substantial updates, which include automated data pulls from the AOP-Wiki 2.0, the integration of tissue-gene network data, and human AOP-gene data by population, semantic mapping and SPARQL endpoint creation, in addition to the presentation of the first publicly available AOP-DB web user interface. Potential users of the data may investigate specific molecular targets of an AOP, the relation of those gene/protein targets to other AOPs, cross-species, pathway, or disease-AOP relationships, or frequencies of AOP-related functional variants in particular populations, for example. Version updates described herein help inform new testable hypotheses about the etiology and mechanisms underlying adverse outcomes of environmental and toxicological concern.

## Abstract (summary)

**Table:** 

Measurement(s)	adverse outcome pathway • gene interactions • Orthologous Gene • chemical gene interactions • molecular pathway • disease gene associations • SNP
Technology Type(s)	digital curation

Machine-accessible metadata file describing the reported data: 10.6084/m9.figshare.14737557

## Background & Summary

There is a need for approaches to understand the biological mechanism of adverse outcomes and human variability in response to environmental chemical exposure. A recent legislation, the Frank R. Lautenberg Chemical Safety for the twenty-first Century Act of 2016^1^, requires the US Environmental Protection Agency to evaluate new and existing toxic chemicals with explicit consideration of susceptible populations of all types (life stage, exposure, genetic, etc.). In addition, on September 10, 2019, EPA Administrator Andrew Wheeler signed a directive that prioritizes efforts to reduce animal testing. In response to this directive, the EPA has developed a 2019 Strategic Plan to Promote the Development and Implementation of Alternative Test Methods Strategies (or New Approach Methodologies (NAMs)) per TSCA Section 4(h)(2)(C). The EPA Adverse Outcome Pathway Database (AOP-DB) is a decision support tool developed by the EPA’s Center for Public Health and Environmental Assessment, which contributes to NAMs (*e.g*. computational toxicology tools) used for TSCA. The EPA Adverse Outcome Pathway Database (AOP-DB) is a database resource that combines different data types (AOP, gene, chemical, disease, pathway, orthology, and ontology) to characterize the impacts of chemicals to human health and the environment^2^, and for the characterization of human genetic susceptibility for the purpose of human health risk assessment^3^. The AOP-DB was originally developed with the primary aim of integrating AOP molecular target information with other publicly available datasets and related toxicological data, to facilitate computational analyses of AOP information. Near term goals for use of the AOP-DB are to address the biological and mechanistic aspects of alternative test methods in terms of the adverse outcome pathway construct to facilitate Integrated Approaches to Testing and Assessment (IATA) for regulatory purposes^4–6^.

Here we present an updated version of the database, AOP-DB v.2, which includes an increased number of adverse outcomes and corresponding key events derived from updated feeds from the AOP-Wiki 2.0 (https://AOP-Wiki.org/), as well as updated chemical, disease, tissue, individual and population level data and ontology information. In this second iteration of the database, we update all code and previously described data from AOP-DB version 1, as well as provide integration with three new data areas: tissue, individual and population level data. We discuss the collaborative semantic mapping efforts for AOP-DB data, and highlight the AOP-DB web user interface, which will be deployed to the public in 2021.

Pittman *et al*.^2^ presented the first version of the AOP-DB which focused on chemical and species-specific analyses. Mortensen *et al*.^3^ developed a computational approach that implements the AOP-DB to integrate mechanistic data associated with an AOP with data capturing human genetic variability and function, for the purpose of characterizing human molecular variation that may impact individual and population level responses to environmental chemicals. The human individual and population level data included in AOP-DB v.2 provide the relevant data sources and organizational structure to envision the approach presented by Mortensen *et al*.^3^. We believe that this work represents a first step in organizing a coherent research program in molecular environmental adverse outcomes. Current and future work will focus on the application and interrogation of these data for case study examples, the development of computational, quantitative AOP (qAOP) models for estimation of chemical-MIE protypes, and machine learning methods to understand human susceptibility and variation in functional response that will inform chemical safety assessment.

## Methods

Data included in the AOP-DB v.2 represent an aggregation of publicly available sources associated with adverse outcome pathways. All data included in AOP-DB v.2 were acquired or generated as detailed below. Online-Only Table 1 lists the current data sources integrated in the AOP-DB v.2. Table 1 lists the actual record count for integrated data within the AOP-DB v.2. For example, 261 supported AOPs in the AOP-DB v.2, as indicated in Table 1, indicates there are 261 unique, expert-derived AOPs that map to one or more unique gene or protein accession numbers. Similarly, for those 261 AOPs, 1029 chemical stressors map chemical ID to AOP ID, underlining a unique AOP-chemical association. The AOP-DB v.2 contains a full list of AOP stressors obtained and updated from the AOP-Wiki 2.0, which is available and updated as part of the OECD supported AOP Knowledge Base (AOP-KB) through e.AOP.Portal (https://aopkb.oecd.org/).

**Table 1:** AOP-DB tables with Summary Count information.

Data Table	Data Type	Count
Gene Info	Unique gene IDs	24609215
Gene Interactions	Pairwise gene interaction scores	4.83E + 08
Species Info	Entrez-supported organisms	26554
Homology Gene	Orthologous Groups	64930
	Taxa supported by ortho groups	605
AOP Info	Supported AOPs	261
AOP gene	AOP gene associations	758
Chemical Info	CTD chemicals	170956
Chemical gene	chemical-gene associations	1206437
AOP Stressor	DTX-AOP associations	654
ToxCast Assays	Assays	406
Pathway Gene	Pathways	110889
	Pathway-entrez links	6412846
Disease Gene	Diseases	24166
	Disease-gene associations	628685
GO Gene	GO terms	26739
	GO-gene associations	1698353
Tissue Networks	tissues	145
	edges	16957011
SNPS	refsnp_ids	2464
	AOP gene associations	5217
SNP frequencies	populations	5
Haplotypes	samples	2504

**Fig. 1:**
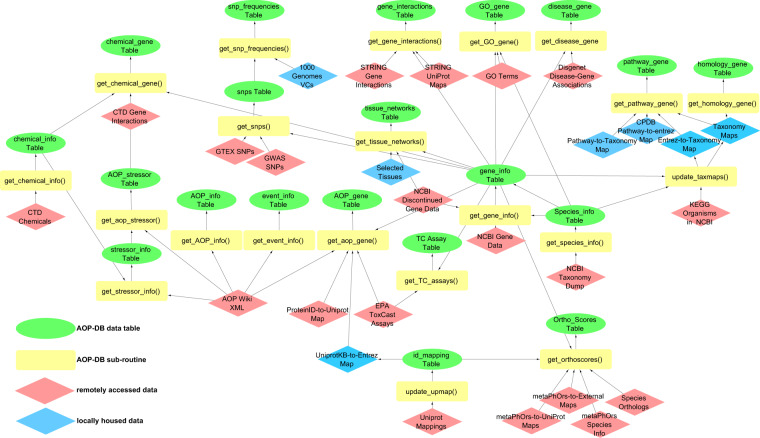
AOP-DB Data Structure. Green, Ovals indicate data tables in the AOP-DB SQL relational database; Blue, Diamonds indicate local, post-processing files necessary, where modified data are stored; Yellow, Rectangles indicate corresponding subroutines necessary to process source data; and Red, Diamonds indicate publicly available, third party source data included in AOP-DB v.2. Arrow edges indicate foreign key relationships.

Methods and included data sources presented here reflect the AOP-DB v.2 as of May 5th, 2021. In addition to regular updates to the AOP-DB, we anticipate that additional data will be added to the database over time. The addition of new data will be announced via the AOP-DB web user interface https://aopdb.epa.gov/, on the AOP-DB ‘Home’ page https://www.epa.gov/healthresearch/adverse-outcome-pathway-aop-database, and the CompTox Chemicals Dashboard (https://comptox.epa.gov/dashboard/), hereafter referred to as the “Dashboard”, on the ‘News’ page, (https://comptox.epa.gov/dashboard/news_info) and ‘Downloads’ (https://comptox.epa.gov/dashboard/downloads) pages, as appropriate. The third-party data sets included in the AOP-DB v.2, and additional details of methods used for any modifications performed in the integration process, are described briefly below and with full details in Fig. 1 and Online-only Table 1.

### AOP-DB v.2

Data selection and collection methods match closely with those used for AOP-DB v.1^2^. AOP-DB v.2 implements the AOP set available with the AOP-Wiki 2.0 (https://aopwiki.org/). AOP-DB v.1^2^ links AOPs to gene and protein accession numbers manually using the concept of the Event-Component using selected gene and protein ontologies, according to the methods described in Ives *et al*.^7^. The AOP-Wiki 2.0 reports the results of an automated ontology mapping process, making automated AOP-gene association updates possible in AOP-DB v.2. AOP-DB v.2 tables are created using MySQL (version 5.7.25) and updated using in-house Perl (version 5.26.3) and Python (3.6) scripts. Data is downloaded and processed from all third-party sources using R (version 3.6.0) and Python (3.6). The AOP-DB v.2 data structure, as described in Fig. 1, illustrates the data integration of the central AOP-gene data with other third-party sources. Figure 1 also indicates how data are integrated across data sets, where subroutines are needed, and local files are stored. The AOP-gene table links chemical-gene (CTD^8^), biological pathway (ConsensusPathDB^9,10^, KEGG Pathways^11–13^, Reactome^14^), disease-gene (DisGenet v.6.0^15^), species homology (Homologene^16^), ToxCast Assay target information^17–20^, tissue-gene mapping (HumanBase^21^, and population Single Nucleotide Polymorphism (SNP) frequency data for functionally relevant AOP gene targets (Ensembl^22,23^; GWAS^24^; GTex^25–27^; 1KGenomes^26,28^). The AOP-DB v.2 SQL database schema is included in Supplemental Fig. 1.

### AOP-Wiki XML import, and mapping of molecular identifiers

AOP-DB v.2 has updated the primary source of AOP information, and now implements the AOP-Wiki 2.0 XML dump, which is updated quarterly. This dump includes all stressor, event, status, and description data associated with AOP’s from the AOP-Wiki 2.0 in XML format. We used the XML package (version 3.98-1.2) in conjunction with xPath (version 3.1) notation to parse the AOP-Wiki 2.0 XML into tabular data for import into the AOP-DB v.2, in order to create a data frame with essential columns. Once organized into tabular format, this processed XML data is separated into AOP_info, AOP_gene, AOP_stressor, stressor_info, and event_info tables. Individual subroutines automate and validate the accuracy of the parsed data at each stage. The AOP_stressor and stressor_info tables, due to somewhat loosely structured submission structure, require manual processing and mapping of chemical ids to chemicals without mapping information, discussed below.

To create the link between AOP’s from the AOP-Wiki 2.0 and gene identifiers in the AOP-DB v.2, which are not supplied in the AOP-Wiki 2.0 XML directly, we map key event information within each AOP containing a biological object, where a biological object is comprised of three ontological components: Process; Object; and Action. When the object term contains a protein ontology (PR) value, this value is considered a “molecular identifier” and is used to map that key event to a corresponding gene identifier (e.g. Entrez, UniProt, etc.).

### AOP-Wiki Stressor tables

After the AOP-Wiki XML is parsed, the AOP-DB stressor tables undergo an additional processing step. The AOP-Wiki XML contains stressor_name, user_term, stressor_id and Chemical Abstract Service Identifier (CASRN). Stressors entered into the AOP-Wiki can include a link to chemical stressors, via the DSSTox Substance Identifier (DTXSID), which maps the stressor to substances registered in the DSSTox database^29^. The chemical DTXSID, a unique substance identifier, provides a link to the Dashboard using the process described in Williams *et al*.^30^. When no DTXSID is provided for stressors imported from the AOP-DB, manual curation to the Dashboard has been performed on individual substances, on a substance-by-substance basis and using available identifiers (e.g. CAS Registry Numbers and chemical names) according to the process described in Grulke *et al*.^31^. A number of issues were addressed in this mapping process for the AOP-DB stressor mapping; for example, the same chemical with different chemical identifiers, mapping to the same substance (e.g. Dexamethasone vs Stressor:492 Dexamethasone); spelling errors (e.g. Tacrorimus versus Tacrolimus); general naming that prevents specific mapping to a DTXSID, for instance, “various hydrocarbons”, that is in no way definitive for a particular substance and cannot be mapped to a DTXSID. It should however be noted that ambiguous substances can map to individual chemical structures, to mixtures or to UVCB chemicals (Unknown or Variable Composition, Complex Reaction Products and Biological Materials), for example, as discussed in Williams *et al*.^30^. The AOP-DB v.2 stressor linkages are provided through the Dashboard (https://comptox.epa.gov/dashboard/chemical_lists/AOPSTRESSORS), and map EPA chemical substance records to the most current list of AOP stressors (last updated 06/30/21).

### AOP-DB Tissue-gene networks

The AOP construct organizes biological and related data from molecular gene targets, both cellular and organ specific, to individual and population levels, so that we can better understand the progression of initiating events of toxicological concern to their benign or adverse outcomes.

The primary aim of the AOP-DB is to organize publicly available data so that users can understand and characterize the biological context of any given AOP. The AOP-DB v.2 addresses the need to understand tissue specific events in an AOP-tissue specific context. Here we implement HumanBase^21^ data, which includes tissue-specific gene networks for 144 human tissues. By combining genome-wide association studies (GWAS) with tissue-specific gene networks, it is possible to develop tissue-specific gene-interaction networks with probabilities representing the strength of the interaction^21^. Using the HumanBase API (https://hb.flatironinstitute.org/api/), we import tissue-gene networks for all 144 tissues included in HumanBase and link to each AOP gene. So, for each AOP gene, 144 individual, tissue-gene networks are added. The number of networks will increase as the number of AOP genes in the AOP-DB increases. The final tabular form of the tissue-gene data contains edge data (Entrez1 and Entrez2) representing gene interactions, with individual edge probability scores. Each edge record has a tissue and query gene field, indicating network relationship.

**Fig. 2:**
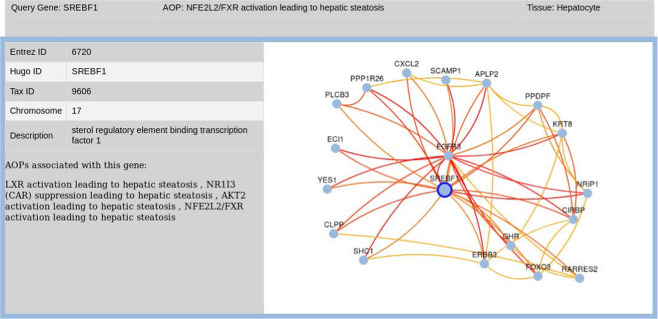
AOP Tissue Network Visualization Tool illustrates the tissue-gene network built with user query for SREBF1 for hepatocyte tissue. Associated AOPs for SREBF1 are listed in the left-hand pane.

The AOP-DB v.2 implements the HumanBase tissue-gene networks in the creation of a tissue-gene visualizer in the AOP-DB web user interface (Fig. 2). This allows users to navigate to AOP relevant genes and tissue-specific results in a real-time network viewer. The tissue-gene network results can be explored for hypothesis generation and, for example, to understand how tissue-specific AOP activity relates to known disease states in the AOP-DB.

### AOP Haplotypes from 1000 genomes single nucleotide polymorphisms (SNPs)

To our knowledge the AOP-DB v.2 is the first tool that incorporates the novel integration of human population level data with AOP information. AOP-DB v.2 incorporates individual and population level data using data from Ensembl^22,23^, ENCODE GWAS^24^, GTex^25–27^, and the 1000 Genomes Project^28,32^, with the goal of identifying functionally important AOP-gene variants. The organization of such functional variants for described AOPs makes it possible to identify potential differences in human susceptibility to adverse outcomes on both an individual and group or population level. AOP-DB v.2 incorporates population level SNP frequencies for AOP-genes for five of the geographic super-populations reported by the 1000 Genomes Consortium (European, S. Asian, E. Asian, African, American^32^) for the Phase 3 data.

**Fig. 3:**
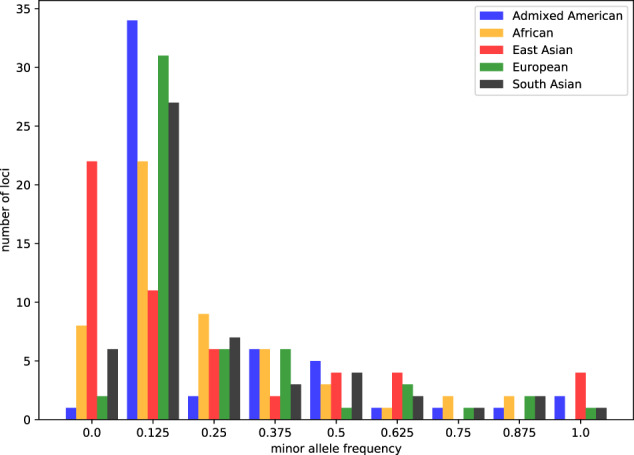
Minor allele frequency (MAF) distribution for SNPs associated with 104 functionally relevant Human AOP-genes for five 1000 Genomes Super populations: American (Blue); African (Yellow); East Asian (Red); European (Green); South Asian (Black).

In addition, we identified SNPs as functionally important from the ENCODE GWAS catalog^24^ and the GTEx portal^25,26^ for each AOP-gene. The SNPs were filtered secondarily using the Ensembl Regulatory Build^23^ to be located in gene regulatory regions. The resulting SNPs were used to request genotypes using the Ensembl REST API’s variation endpoint for each SNP for each AOP-gene. With these data we can construct individual AOP-gene haplotypes. These data are stored in the Haplotype table of AOP-DB v.2. The resulting functional AOP-gene list for each AOP was then screened using 1000 genomes samples to identify population frequencies of functionally relevant AOP gene SNPs and AOP haplotypes. Figure 3 illustrates the minor allele frequencies (MAF) observed for 104 functionally relevant Human AOP-gene SNPs. These multi-allelic, functional AOP-gene haplotypes can be used for further inquiries to determine levels of variation and significant differences in outcome across population groups.

### AOP-DB web-based user interface

The EPA AOP-DB user interface is delivered via a graphical web user interface (coded in Java and React), an application programming interface (API; coded in node.js) and a relational database (MySQL and MariaDB). The AOP-DB web user interface, publicly available at https://aopdb.epa.gov/, has been developed with the goal of providing both data accessibility and data visualization according to the FAIR Principles^33^ ^2019b^. Basic search functionality of the AOP-DB web user interface allows users who prefer to interact with the web user interface to easily navigate, organize and download AOP and related data to their desktop.

**Fig. 4:**
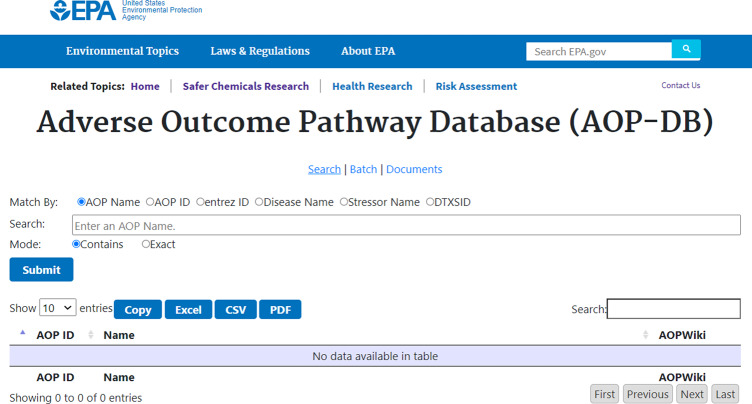
AOP-DB search page illustrating “Basic Search” task bar with accepted user input types and download file specificity.

Basic functionality includes a search interface (Fig. 4), batch query tool, and downloads page. The AOP-DB system has been modified for EPA standard visualization as well as integration with current Office of Science and Information Management (OSIM) security protocols for EPA internet hosting. At present, data updates to the AOP-DB occur approximately every 6 months. Initial AOP-DB data visualization has been optimized and includes the following queries: AOP-gene, AOP-disease, AOP-pathway, and AOP-chemical. Search box functionality has also been extended to include autofill processing for inputs. Download capabilities have been extended to include user column select and scroll features, including data download in .csv and .xml, or other useful formats. The ‘Documents’ page links to the AOP-DB v.2 User Manual, which describes all current search functionality enabled for the user interface in detail, and includes sections on maintenance of new records and updates, how content is selected, query tips, and frequently asked questions.

### Research description framework mapping of the AOP-DB data tables

**Fig. 5:**
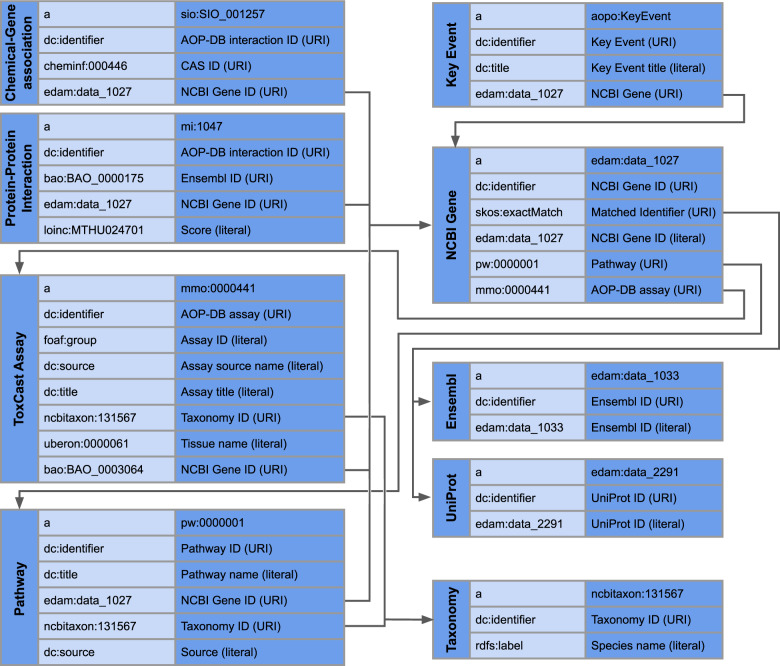
AOP-DB Semantic Mapping using the Resource Description Framework (RDF) illustrating Chemical-Gene Interaction, Protein-Protein Interaction, ToxCast Assay, and Pathway tables.

Semantic mapping using a Research Description Framework (RDF)^34^ was performed for four AOP-DB tables^35^ as part of an ongoing, international collaboration between the US EPA, OpenRiskNet (https://openrisknet.org/) and researchers at the Department of Bioinformatics – BiGCaT, Maastricht University (http://www.bigcat.unimaas.nl/). Figure 5 illustrates the AOP-DB RDF tables for Chemical-Gene Interaction, Protein-Protein Interaction, ToxCast Assay, and Pathway, and each table’s secondary (and tertiary) keys. A link to the AOP-DB SPARQL endpoint and the workflows generated using these mappings is provided by the OpenRiskNet e-infrastructure (https://openrisknet.org/e-infrastructure/services/147/).

## Data Records

Online-only Table 1 describes all the individual data source integrated in the AOP-DB. Data sources are organized according to their biological category, with a short description of the data, along with any manipulations performed, and the URL. The AOP-DB v.2 data frame with all corresponding table data and custom code has been uploaded to a single collection entitled “The Adverse Outcome Pathway Database (AOP-DB) version 2.0”^36^. The files contained in this collection include the most recent SQL data structure for the AOP-DB v.2, all custom code, tables, and corresponding data categories and keys to create the backend of the database.

## Technical Validation

The main quality objective for AOP-DB is ensuring that data included in the data set accurately reflects the data obtained from the raw data source. Data sets considered for curation in the AOP-DB are limited to publicly available, peer-reviewed datasets that have been subjected to rigorous quality control and peer review in their own right. Quality assurance measures to validate the data have focused on ensuring that data records were transcribed accurately from the original data source and represented appropriately in the repository. As a research product of the United States Environmental Protection Agency Office of Research and Development (ORD), the AOP-DB has a Quality Assurance Project Plan (QAPP) which describes each individual data source integrated in the database, how it is implemented, stored and modified. QAPPs describe the necessary quality assurance and quality control measures needed to produce results that meet stated performance criteria. ORD QAPPs are peer-reviewed, approved by management, overseen by a quality assurance manager, and subject to periodic QA and performance quality checks. Figure 1 illustrates, for each data source, each step in obtaining the data from the third-party source, any subroutine that has been created to modify the data, and including any stored local files. In all cases, custom scripts were written to identify and correct any errors in the process of migrating data from source formats into the MySQL database (*e.g*. duplicated entries).

With public deployment of the AOP-DB v.2, data updates occur approximately every 6 months. Additional, periodic updates will be made to the production version of the database when substantial changes have been made requiring a version update. Changes to the development version of the database will be ongoing and as new, and relevant data become available.

## Usage Notes

Though data included in AOP-DB v.2 can be used in many ways in future analyses, users should be aware of limitations of the data set, and appropriate usage of the data. The AOP-DB v.2 incorporates only those AOPs that have molecular identifiers. Many AOPs found in the AOP-Wiki 2.0 do not associate with gene or protein molecular identifiers. This may be due to the nature of the biology described or the state of the science for a particular research area. Users should also be aware that AOP data obtained from the AOP-DB v.2 may be under various levels of review (e.g. Status or SAAOP Status “Do not cite”, or “Included in OECD Workplan”, for example) or the AOP may simply be incomplete. This is due to how AOPs are entered into the AOP-Wiki 2.0 at this time. Additionally, because an AOP may be developed with reference to a particular species, the AOP-DB v.2 reports the gene/protein accession for that species and maps to orthologous gene pairs in humans or other species orthologs when possible, without indication of relevance.

## Supplementary information

Supplementary Figure 1

## Data Availability

All custom code created to process of manipulate external datasets in the construction or subsequent update of the AOP-DB v.2 relational database tables are made publicly available by the U.S. Environmental Protection Agency, Office of Research and Development (ORD)^36^.

## Online Only Table

**Online Only Table 1:** Additional data sources included in the AOP-DB.

*Biological Category*	*Data Source*	*Description*	*Processing*	*URL*
Gene	NCBI Gene	This source supplies all NCBI entrez genes in the gene info table with associated gene information such as name, symbol, location, etc.	The latest gene_info and gene_history files are pulled from the NCBI GENE FTP. gene_history contains all deprecated entrez which are used to remove entries from the gene_info table. No other processing takes place	ftp://ftp.ncbi.nlm.nih.gov/gene/DATA/
	STRING	This source gives protein-protein interaction data for the gene-interactions table. Each record from these networks is stored with an entrez1, entrez2, and an interaction score	STRING files are downloaded (no api.or ftp) and proteins are mapped to entrez via unitprot. (UPKB2entrez). No other processing takes place	http://string-db.org/cgi/download.pl
Taxonomy & Orthology	NCBI Taxonomy	All taxa available from NCBI, including nomenclature info and divisions. This data is used to fill the species_info	The taxonomy data are pulled from the NCBI FTP. No other processing takes place.	ftp://ftp.ncbi.nlm.nih.gov/pub/
	Homologene	Constructs and stores putative homology groups and contributes and ortho group number, tax id, entrez id to the homology gene table	The homogy_gene file is pulled from the NCBI FTP. No processing takes place.	ftp://ftp.ncbi.nlm.nih.gov/pub/HomoloGene
	KEGG Orthology	This database of functional orthologs contributes ortho group ids, tax ids, and entrez ids describing an orthologous group to the homology gene_table	The latest ortholog data is pulled from the KEGG “R” Package KEGGREST. Kegg Gene IDs are mapped to entrez. No Processing takes place.	http://www.kegg.jp/kegg/rest/keggapi.html
	metaPhOrs	This database of phylogeny based orthologs contributes ortho group ids, taxonomy ids, and entrez ids to the homology gene table, describing orthologous groups.	Mappings and ortholog data for a manually curated set of species are pulled from the phylomedb FTP. Only protein mappings from SwissProt and TrEMBL are considered. Protid IDs are mapped to entrez. Where protid2 is unmapped or empty, it is mapped to entrez = 0. No other processing takes place	ftp://phylomedb.org/metaphors/
AOP	AOP-wiki	This is a collaborative set of AOPs regularly updated with new details or new Adverse Outcome Pathways. This source contributes to the central AOP_info tables and the AOP_gene tables, supplying AOP names, key events, descriptions, and information used to map key events to genes.	The latest aop wiki xml dump is downloaded from the AOPWiki downloads page. No other processing takes place	https://aopwiki.org
Chemical	CTD	This source is a manually curated database of chemical information, including many modules. The module of interest for the AOPdb is the chemical gene interactions module, which contributes chemical names and ids to chemical info, as well as the chemical gene interactions with contextual information to the chemical gene table.	The latest chemical tables and chemical gene interactions are downloaded from CTD website. No Processing takes place.	http://ctdbase.org/downloads/
	AOP-wiki	In addition to being the source of AOPs for the AOPdb, this source also adds chemical stressors related to the MIE of each AOP. This data contributes chemical names, as well is DTXSIDs, casrns, or other chemical ID when available.	Latest AOPWiki XML dump is downloaded. All aop, stressor, and key event information is merged together. No other processing takes place	https://aopwiki.org
	ToxCast	This is a collection of high-throughput screening assays for chemicals that contributes assay identification information and assay context information as well as gene target information in the form of entrez ids.	ToxCast Assay data is loaded from a static file. No processing takes place	ftp://newftp.epa.gov/comptox/High_Throughput_Screening_Data
Pathway	KEGG Pathways	This source is a collection of biological molecular interaction pathways that supplies entrez ids and pathway names and ids, linking gene components to the pathways in which they are involved.	Pathway data is accessed through the KEGG API, KEGGREST (version 1.24.0). KEGG gene IDs are mapped to Entrez ID. Entries without an Entrez are dropped. No other processing takes place.	http://www.kegg.jp/kegg/rest/keggapi.html
	Reactome	This curated and peer-reviewed source of molecular pathways supplies entrez ids and their linked pathways to the pathway_gene table of the AOPdb	Reactome data is loaded, and Tax_ids are mapped using species name and species ID. No other processing takes place.	http://www.reactome.org/pages/download-data
	ConcensusPathDB	This source brings together pathway and interaction data from 32 public resources and supplies entrez ids and pathway ids that link genes to biological pathways for the pathway gene table	CPDB data is loaded from a static file. Tax_ids are added by mapping entrez ID to species ID. No other processing takes place.	http://consensuspathdb.org/
Disease	DisGeNET	This database compiles different data, both curated and inferred from models, and supplies multiple downloadable tables relating genes and variants to the diseases in the database. The AOPdb uses DisGeNET’s gene-disease association table, adding all fields to the disease-gene table. These include disease name and id, entrez id, and a score for the association based on its sources.	The latest disese gene associations are downloaded via the Disgenet website. No other processing takes place.	http://www.disgenet.org/downloads
Ontology	NCBI Gene	In addition to being a source of taxonomy info and gene info, NCBI Gene supplies gene ontology information. This supplies gene ontology terms and any related entrez ids to the GO gene table.	The latest gene2go (gene GO mapping file) is downloaded from the NCBI FTP site. No other processing takes place.	ftp://ftp.ncbi.nlm.nih.gov/gene/DATA
Tissues	HumanBase	This API is used to pull tissue specific gene interaction network from HumanBase. The data imported into the tissue networks table in the AOPdb include entrez1 and entrez2 fields to construct edges, as well as a probability score indicating the strength of the modeled gene interaction.	Tissue networks are independently retrieved through the HumanBase API for all genes and all tissues. Tissue-gene combinations without edges are dropped. Genes are compared to NCBI’s list of deprecated and entrez ID is updated. If an edge has a deprecated gene it is dropped, if it is changed the tissue_networkgene is updated. No other processing takes place.	https://hb.flatironinstitute.org/api/
Haplotypes	1000 Genomes	This is a collection of variant data for individuals from a multitude of populations. This source contributes snp frequencies for each function snp in the snps table for each of 5 1000 Genomes major populations.	1000 Genomes Phase 3 static VCF files are downloaded and stored locally. Using VCF tools we filter the data set to SNPs retrieved from GWAS and GTEX. No other processing takes place.	
	Ensemble	This API, allowing access to ensembl’s gene and variant information, is used to get genotype data for each individual sample from the 1000 Genomes project. These data are used to construct haplotypes for each AOP and find differences in haplotype frequencies within and between populations.	Ensembl Regulatory regions are loaded from a static file and filtered by AOP-DB SNPs. Genotype, minor allele, variant id are then downloaded for each loci using the Ensembl REST API. No other processing takes place.	https://rest.ensembl.org/
	GWAS Catalog	This is a source used to filter SNPs into snps of interest for variant analysis in different populations. Functional snps are specifically targetd. It, along with GTEx, supplies refsnp ids for these variants as well as contextual information.	SNPs are pulled from the static file GWAS Catalog 1.0.2. No other processing takes place.	https://www.ebi.ac.uk/gwas/
	GTEx	This is a source used to filter SNPs into snps of interest for variant analysis in different populations. Functional snps are specifically targetd. It, along with GWAS Catalog, supplies refsnp ids for these variants as well as contextual information.	SNPs are pulled from the static file Gtex V7 Single Tissue cis eQTL. No other processing takes place.	https://www.gtexportal.org/home/
