# Supplementary figures and images for: The 2021 update of the EPA’s adverse outcome pathway database
Source: Sci Data. 2021 Jul 12;8:169. doi: 10.1038/s41597-021-00962-3 (PMC8275694; doi:10.1038/s41597-021-00962-3)

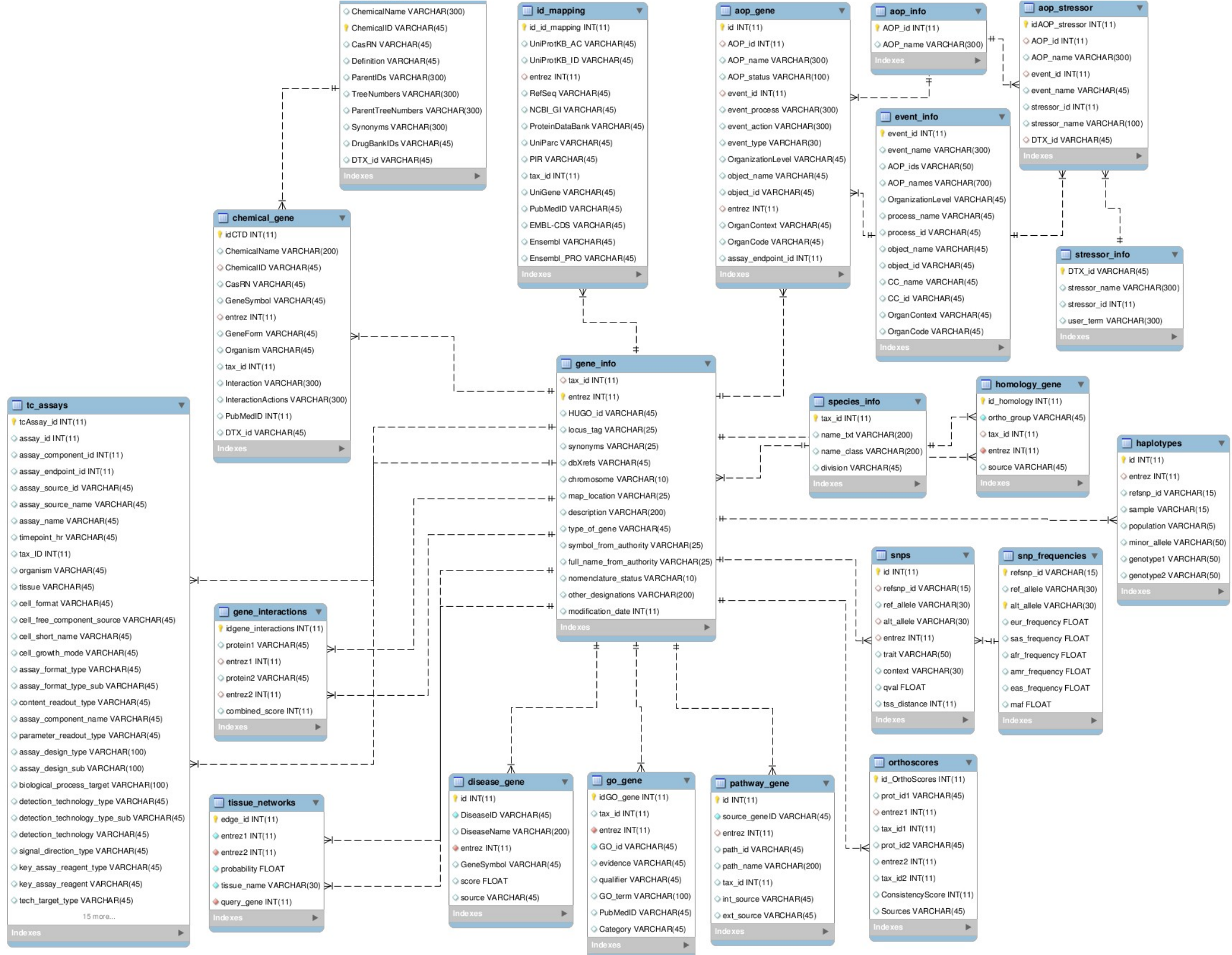

Supplement: Supplementary file 1 — Supplementary Figure 1 [file 41597_2021_962_MOESM1_ESM.pdf]
